# Si-Mg isotopes in enstatite chondrites and accretion of reduced planetary bodies

**DOI:** 10.1038/s41598-020-57635-1

**Published:** 2020-01-27

**Authors:** Jinia Sikdar, Vinai K. Rai

**Affiliations:** 1 Physical Research Laboratory, Ahmedabad, 380009 India; 2 School of Earth and Space Exploration, Arizona State University, Tempe, AZ 85281 USA

**Keywords:** Geochemistry, Geochemistry

## Abstract

Among the primitive meteorite classes, Enstatite Chondrites (EC) are believed to share a common origin with the Earth due to its close similarity with terrestrial mantle (Bulk Silicate Earth, BSE) for numerous isotope systematics. Si isotopes are an exception to this trend and the large δ^30^Si difference of ~0.3‰ between bulk EC and BSE has been used to argue against any major contribution of EC like planetary materials in Earth’s accretion. However, Si possess a bimodal distribution among silicate and metallic fractions of EC because of its formation under highly reducing conditions. Based on high precision Si isotope analyses in micro-milled phase separates of EH3 chondrites, here we report the presence of significantly light Si isotopes in EC-metals (δ^30^Si ≥ −6.94 ± 0.09‰, Mg/Si = ~0.001) whereas its silicate phases are isotopically heavier (Av. δ^30^Si_EC-silicates_ = −0.33 ± 0.11‰, Mg/Si = ~1.01) and closer to BSE (δ^30^Si_BSE_ = −0.29 ± 0.08‰). We discuss the origin of the observed Si isotope heterogeneity in terms of gas-solid interaction processes associated with metal-silicate condensation at high C/O environment (~0.83). Although the elevated δ^30^Si of BSE compared to chondrites is consistent with earlier conclusions that lighter Si has partitioned into Earth’s metallic core, our results indicate that the super-chondritic Si isotope composition of BSE does not reflect the sole consequence of high temperature-pressure core and mantle equilibration in a deep magma-ocean. Instead, Si along with Mg isotope analyses carried out in the same aliquot of EC micro-phase separates suggest that processes such as metal-silicate Si isotope fractionation at reduced nebular environment and vapor loss of lighter Si isotopes during planetary volatilization were also influential in establishing the Si isotope composition of terrestrial mantle.

## Introduction

Searching for the basic building blocks of inner planets of the Solar System has remained one of the long-standing enigmas in geo- and planetary-sciences. On the ongoing debate, terrestrial planets are argued to have accreted from different classes of chondrites (*i.e*. carbonaceous (CC), ordinary (OC), and enstatite chondrites (EC)), achondrites or no longer extant meteorites combining in variable proportions^1–5^. It is generally assumed that all planetary bodies were formed from primitive solar nebula that had carbonaceous chondritic composition because of the similar relative elemental abundances of CI chondrite as solar photosphere for all except the most volatile elements^6^. As far as the Earth is concerned, its refractory lithophile element budget can be best explained by carbonaceous chondrite building blocks^1,3^. However, the presence of significant isotope anomalies in CC relative to terrestrial mantle (Bulk Silicate Earth, BSE) cast doubt on any major involvement of CC like planetary bodies in Earth’s formation^7^. Compared to carbonaceous chondrites, the isotope anomalies are less extensive in ordinary chondrites whereas enstatite chondrites display resolvable yet the least nucleosynthetic/stable isotope differences relative to BSE for a large number of isotope systematics such as O, S, N, Mo, Ru, Ni, Cr, Ti, Fe, Os, Nd, Ca, Zn, Sr, and Mg^8–22^, indicating a possible genetic link between EC and the Earth^4^. Silicon isotopes have remained one of the exceptions to this observation and the large δ^30^Si difference between bulk enstatite chondrites and BSE of ~0.3‰ has called for arguments against any major involvement of EC like planetary bodies in Earth’s accretion^23^ (where δ^30^Si expresses the per mil variation in Si isotope ratios of the sample relative to NBS-28 standard). Moreover, due to formation under highly reducing conditions, enstatite chondrites have a unusual mineralogy composed predominantly of FeO-poor enstatite (MgSiO_3_, typically with Fe content of <1%), Si-bearing Fe–Ni metal, free silica, troilite (FeS), and a variety of rare sulfide and nitride minerals such as MgS, CaS, MnS, TiN and Si_2_N_2_O etc, which are not usually reported from terrestrial rock samples^24^. Apart from mineralogical differences, the budget of elements such as Al, Ca, Si, Mg in EC^25^ is also not compatible with the chemical composition of BSE, which further speaks against enstatite chondrite composition of Earth’s building blocks.

**Figure 1:**
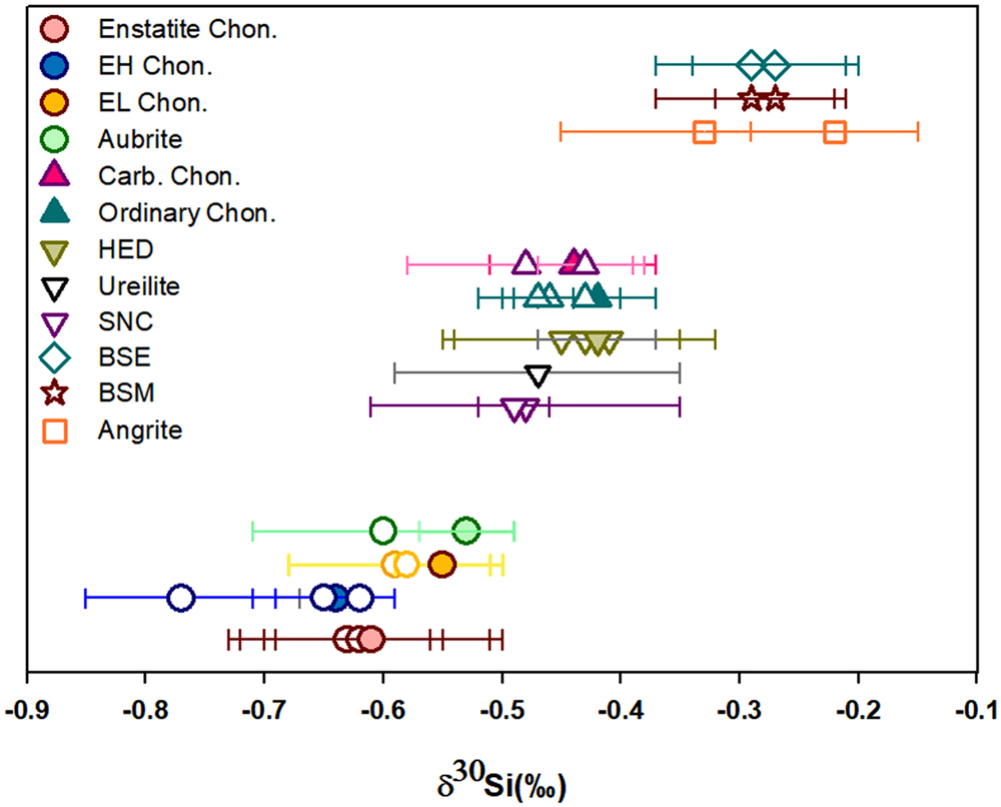
Plot showing the three broad groups of planetary materials distinguished based on Si isotope variations. The filled legend symbols are the average Si isotope composition of individual meteorite groups determined in present study and the unfilled symbols represent average δ^30^Si of meteorites obtained from published literature^26,30–35^.

After oxygen and iron, silicon is the third most abundant element of the Earth and one of the fundamental mineral-forming elements of terrestrial planets. Since Si is involved in almost every major geo-(cosmo)-chemical processes, the distribution of Si isotopes among chondrites and BSE places important constraints to decipher the nature and evolution of materials that dominated the terrestrial planet forming region. In the present study, we have analyzed high precision Si isotope composition of several bulk meteorites including five CC, ten OC, six EC, and eight HED (Howardite, Eucrite, Diogenite) clan of meteorites (Table S1 in supplementary text). Our isotope results are consistent with previous conclusions that bulk enstatite chondrites display the largest δ^30^Si offset from BSE compared to any other primitive meteorites. From data-set obtained in this study and literature values^26–35^, it is evinced that all planetary bodies can be grouped into three broad categories based on δ^30^Si variations (Fig. 1): (i) Enstatite chondrites and their differentiated counterparts ‘the Aubrites’ have resolvably lighter Si isotope composition (δ^30^Si_Enstatite-meteorites_ ≤ −0.53 ± 0.04‰); (ii) Ordinary-Carbonaceous chondrites, HEDs, Ureilites and Martian meteorites have intermediate composition (δ^30^Si ranges from −0.52 to −0.33‰), (iii) Terrestrial rocks, Lunar samples and Angrites are isotopically heavier (δ^30^Si ≥ −0.29 ± 0.08‰). Uncertainties associated with isotope data are given in 2 standard deviation (2 SD) of the mean throughout the manuscript.

The heavier Si isotope composition of BSE compared to undifferentiated chondrites has been most popularly explained by the partitioning of light Si isotopes into Earth’s metallic core^27,30,31^. This is corroborated by geochemical (superchondritic Mg/Si ratio of BSE^1,2^) and geophysical constraints (density deficit of Earth’s core relative to pure Fe-Ni alloy^36^), which requires the presence of ~6–10 wt% light elements in the core^36^. Since the density of Earth’s inner core can not be matched by a metal alloy with more than 5 wt% Si for all reasonable core temperatures, it is speculated that the core might host other light elements apart from Si^37^. In absence of direct samples from Earth’s core, the non-chondritic Si isotope composition of BSE has been extensively used to indirectly estimate, via mass balance, the Si content of the core^27,28,30,31^. However, the calculated amount of Si using the above approach varies with the choice of chondrites taken as a proxy for undifferentiated bulk Earth composition. For example, the δ^30^Si difference of ~0.15 ± 10‰ or more between BSE and ordinary/carbonaceous chondrites suggests the presence of atleast 12wt% Si in the core^30,31^ for continuous Earth accretion model. On the other hand, the relatively larger δ^30^Si offset between BSE and EC (Δ^30^Si_BSE-EC_ = δ^30^Si_BSE_ − δ^30^Si_Bulk EC_ = ~0.34‰) demands ~26 wt% Si in the core for enstatite chondrite Earth model^23^, which is unrealistically high and cannot explain core’s geophysical properties. Based on these estimates, it has been concluded that a major portion of the Earth is made up of a combination of ordinary-carbonaceous chondrites (LL, CI, CO) and a maximum of 15% enstatite chondrites^23^.

Such mass balance calculations to determine Si content of the core assume that different proportions of light and heavy Si isotopes were accumulated in the core and mantle of the Earth during high temperature-pressure metal and silicate equilibration processs generated by core formation above ~2500 K. However, a recent experiment has determined that the metal-silicate equilibrium isotope fractionation factor for Si at high temperature-pressure environment is rather low (−1.48 ± 0.08‰ and −1.11 ± 0.14‰ at 1450 °C and 1750 °C, 1 GPa respectively)^38^. Also, the heavy δ^30^Si of angrites cast doubts on the association of Si isotope fractionation with core formation because the redox conditions required for sufficient Si partitioning into metals does not match that of angrite parent bodies^34,35^. Based on the observed correlation between δ^30^Si and Mg/Si among varied planetary materials, it has been interpreted that nebular fractionation during forsterite condensation (and not core segregation) has caused significant Si isotope fractionation in inner solar system^35^. An alternate hypothesis for the heavy δ^30^Si of angrites and BSE is vapor loss of lighter Si isotopes during early accretionary impacts^22,34^. Therefore, the most influential cause for the observed δ^30^Si variations among planetary-scale objects has remained debated, which needs to be first addressed before making any concluding remarks on the building blocks of Earth from the non-chondritic Si isotope composition of BSE.

In this study, we have attempted to understand the origin of Si isotope composition of terrestrial planets by studying Si (along with Mg) isotope distribution in distinct micro phase-separates of enstatite chondrites, whose reduced mineralogy indicates their formation in the inner region of proto-planetary disk possibly where terrestrial planets had accreted^39^. Moreover, ECs are the only naturally available rock samples where Si is found to be substantially partitioned among its metallic and silicate phases due to the unique siderophilic behavior of Si at low oxygen fugacity^40^. The common Si bearing metals in EC include Kamacite (Fe-Ni alloy), perryite ((Ni,Fe)_8_(Si,P)_3_) and schreibersite ((Fe,Ni)_3_P), containing an average 2.6, 12 and 0.1 wt% Si respectively^24,40^. On the other hand, FeO-poor enstatite (MgSiO_3_) is the most dominant silicate mineral in EC^24^ and hence it is the key phase that can provide important information on conditions and processes responsible for formation of such reduced planetary bodies.

## Samples and Results

Enstatite chondrites are generally grouped into Fe-rich EH and Fe-poor EL chondrites based on Fe content and are further classified into EH3, 4, 5 and EL3, 6 depending on the degree of thermal alteration, 3 being the least altered and 6 being the most equilibrated^41^. Our Si isotope analysis of six bulk enstatite meteorites (four EH3, one EL6, one aubrite) are in agreement with an earlier study^26^, where it is observed that EH chondrites (Av. δ^30^Si = −0.64 ± 0.03‰) have lighter Si isotope composition than EL chondrite (δ^30^Si = −0.55 ± 0.04‰) and aubrite (δ^30^Si = −0.53 ± 0.04‰). However, a heavier Si isotope composition of EL6 chondrite with δ^30^Si = −0.41 ± 0.08‰ has been also reported^35^. All these data reflect the existence of wide spread Si isotope heterogeneity among different types of EC, which is possibly associated with different proportions of metal and silicate content in them. Two independent studies have shown that the non-magnetic fractions of EC have heavier Si isotope composition compared to its bulk meteorite composition^23,26^. For example, δ^30^Si of bulk EH3 chondrites Qingzhen and Sahara 97158 was determined to be −0.82 ± 0.11‰ and −0.55 ± 0.04‰ whereas its corresponding silicate fractions have δ^30^Si = −0.45 ± 0.11‰ and −0.41 ± 0.03‰ respectively^23,26^. Such observations further hint towards an inverse relationship between δ^30^Si and metal content of such meteorites with the possibility of light Si isotope enrichment in metallic phases of EC^26^. However, δ^30^Si of metals from EH3 chondrites has not been precisely determined so far and therefore the extent of Si isotope heterogeneity in such meteorites has remained largely unknown. In one of the above-mentioned studies, ~2–5 g of meteorite samples were first ground to fine powder in agate mortar, from which ~10 mg of non-magnetic fractions (separated using hand magnet) were considered for further chemical processings^26^. In another study, the non-magnetic fractions were first separated using a hand magnet followed by hand-picking of enstatite grains under microscope^23^. Since metals occur as finely disseminated phases throughout EH3 chondrites (Fig. S1 in Supplementary text), it is difficult to sample purest end members of magnetic and non-magnetic phases from EC using hand-magnets, especially when ≥1 g sample has been already homogenized.

To avoid the sampling of metal inclusions in silicates and vice-versa, we have adapted a different analytical approach (detailed in Methodology section) involving micro milling of distinct metallic, silicate and matrix fractions from three EH3 chondrites (PCA 91461, LAR 06252, MIL 07028). At first, polished thick sections of EH3 chondrites were mineralogically mapped using Electron Probe Micro Analyzer (EPMA) and the chemical composition of target phases were quantitatively determined using WDX. The location of the pre-characterized silicate and metallic phases were then identified under optical microscope and precisely micro-milled using New Wave Micromill machine equipped with a drill mounted on a video microscope assembly and a set of motorized stages. Following sample digestion and cation exchange chromatography, high precision Si isotope compositions were determined using Thermo Neptune Plus MC-ICPMS in i) 14 metal enriched grains (Av. Mg/Si = ~0.002 ± 0.005), ii) 12 silicate grains (Av. Mg/Si = ~1.01 ± 0.05), and iii) several silicate-enriched finely disseminated mix phases composed of random proportions of metals and sulfides (referred here as “matrices”). Additionally, Si isotope compositions of metal and non-metallic fractions from two more EH3 chondrites (Y-691 and Parsa) separated via density segregation were also determined in this study. Emphasis has been given on the least equilibrated EH3 chondrites for micro-scale isotope analyses because they preserve the pristine composition of solids that aggregated in reduced inner nebula.

**Figure 2:**
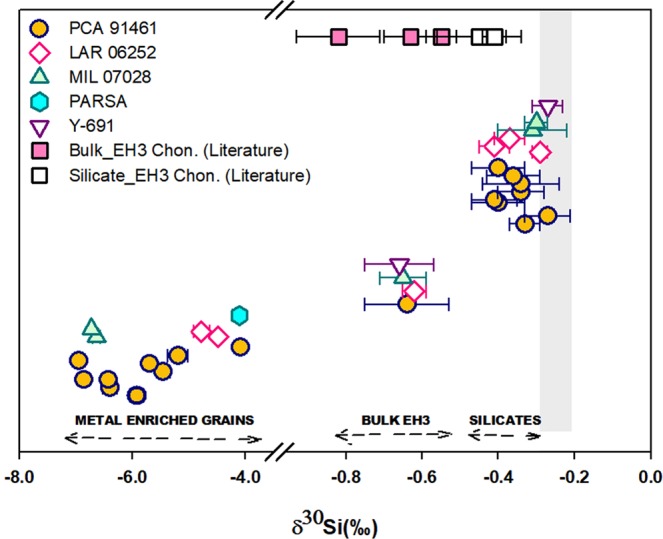
Plot showing the variation of Si isotopic composition among different micro-phase separates of EH3 chondrites. The metallic fractions of EC are enriched in significantly lighter Si isotopes whereas the silicate fractions have relatively heavier δ^30^Si compared to bulk EH3 chondrites. The established range of Si isotope composition for Bulk Silicate Earth (δ^30^Si_BSE_) is represented by grey vertical bar^31,33^. Error bars represent 2 SD of the mean. Literature values of bulk EH3 chondrite^23,26,30^ and their silicate separates^23,26^ are plotted on the top for comparison.

Si isotope data from micro-phase separates of unequilibrated enstatite chondrites are summarized in Table 1. Our results reveal considerable Si isotope heterogeneity among phase separates of EH3 chondrites with the metal-enriched phases being characterized by significantly light Si isotope composition (δ^30^Si_EC-metals_ ranges from −6.94 ± 0.09‰ to −4.08 ± 0.05‰). The lightest δ^30^Si of metal grains determined in this study is in accordance to *in situ* Si isotope data reported from metallic components of enstatite achondrites – ‘the aubrites’^29^. Compared to metals, the silicate phases of EC were determined to be substantially heavier with δ^30^Si ranging from −0.41 ± 0.06 to −0.27 ± 0.06‰. Although on a lighter side, the average Si isotope composition of EC-silicates (Av. δ^30^Si_EC-Silicates_ = −0.33 ± 0.11‰) is close to the established δ^30^Si of Bulk Silicate Earth (δ^30^Si_BSE_ = −0.29 ± 0.08‰^33^, Fig. 2). The Si isotope composition of silicate-enriched  matrices varies from δ^30^Si = −0.58 ± 0.07‰ (almost no metals) to −1.40 ± 0.06‰ depending upon the mixing proportions between isotopically heavier silicates and lighter metals in the target micro-milled phases of EH3 chondrites. Overall, the light Si isotope enrichment of bulk enstatite meteorites (Av. δ^30^Si = −0.61 ± 0.11‰, n=6) compared to CC-OC (−0.43 ± 0.06‰) can be broadly attributed to the presence of isotopically light Si in its metallic phases. The results obtained in this study reinforce theoretical calculations^27,42^ and metal-silicate partitioning experiments^38^, which have demonstrated that metals are consistently enriched in lighter Si isotopes relative to silicates at low oxygen fugacity. Such fractionation probably arises due to different bonding environment of Si in metals and silicates^27^ as light isotopes get preferentially partitioned into metals characterized by weaker ‘Si-Fe’ metallic bonds and the silicates (with stiffer ‘Si–O’ covalent bonds) get enriched in heavier Si isotopes.

**Table 1:** Summary of silicon isotope results from micro-phase separates of unequilibrated enstatite chondrites (EH3) relative to NBS-28 standard.

Sample ID	Range of δ^30^Si (‰)	Average δ^30^Si (‰)	Mean Mg/Si	Mean Fe/Si	N
**Bulk EH3 chondrites**					
PCA 91461	—	−0.64 ± 0.12	—	—	8
LAR 06252	—	−0.62 ± 0.03	—	—	3
MIL 07028	—	−0.65 ± 0.06	—	—	2
Y-691	—	−0.66 ± 0.09	—	—	4
**Metal enriched phases**					
PCA 91461	−6.94(±0.09) to −4.08(±0.05)	−5.84 ± 1.7	0.001	32.23	9
LAR 06252	−4.77(±0.14) to −4.48(±0.05)	−4.66 ± 0.3	0.002	32.71	2
MIL 07028	−6.72(±0.03) to −6.63(±0.07)	−6.69 ± 0.1	0.001	32.18	2
Parsa	—	−4.11(±0.13)		—	1
**Silicate-enriched matrices**					
PCA 91461	−0.85(±0.05) to −0.58(±0.07)	−0.65 ± 0.18	—	—	9
LAR 06252	−1.12(±0.12) to −0.69(±0.07)	−0.87 ± 0.42	—	—	4
MIL 07028	−1.40(±0.06) to −0.98(±0.13)	−1.14 ± 0.42	—	—	3
**Silicate grains**					
PCA 91461	−0.41(±0.06) to −0.27(±0.06)	−0.34 ± 0.11	1.01	0.005	7
LAR 06252	−0.41(±0.04) to −0.29(±0.02)	−0.36 ± 0.11	1.01	0.008	3
MIL 07028	−0.31(±0.09) to −0.28(±0.03)	−0.29 ± 0.06	1.02	0.005	2
Y-691	—	−0.27(±0.04)	—	—	1
Average EC-silicates	−0.33 ± 0.11	—	—	—	—
**Fitoussi and Bourdon, 2012**					
Bulk EH3 Chondrite, Sahara 97158	—	−0.55 ± 0.04 (2SE)	—	—	41*
Silicate-EH3 Chondrite, Sahara 97158	—	−0.41 ± 0.03 (2SE)	—	—	12*
**Savage and Moynier, 2013**					
Bulk EH3 Chondrite, Qingzhen	—	−0.82 ± 0.11	—	—	13*
Silicate-EH3 Chondrite, Qingzhen	—	−0.45 ± 0.11	—	—	5*

Uncertainties are given as 2 SD of the mean. The Mg/Si and Fe/Si values of silicate and metallic phases have been calculated based on results from WDS analyses.‘N’ refers to the number of individual micro-milled phases where isotope analyses were performed. Si isotope data of  bulk EH3 chondrites and its magnetic separates from literature are cited for comparison, where ‘N*’ refers to the number of repeat analyses.

The robustness of our micro-milling and mass spectrometric technique was established by analyzing the Si isotope composition of several meteorites obtained in epoxy-mounted form from ANSMET. The bulk rock representatives of these samples were acquired through micro-milling an entire surface of their polished section through raster-scan mode of drilling. Our Si isotope data of bulk meteorites sampled through two different techniques, i.e., powdering pieces of meteorites in diamonite mortar-pestle and raster scale micro-milling of their polished mounted sections, agree well with literature values (Table S1, SOM) and none of the samples were found to deviate from the mass-dependent terrestrial fractionation line. Secondary Si standard ‘Diatomite’ and USGS rock standard ‘BHVO-2’ were routinely analyzed during the course of individual measurement sessions to monitor the accuracy of isotope data.

## Discussion

From Fig. 1, it is evinced that the Si isotope composition of CC, OC, HEDs, Ureilites, SNCs etc. varies across a narrow range whereas bulk enstatite chondrites are significantly lighter. Also, ECs display extensive refractory lithophile element fractionation as the Mg/Si ratio of EH = 0.73 and EL = 0.88 is lower compared to Mg/Si ratio of ~1 for ordinary and carbonaceous chondrites^25,35^. The correlated variation between δ^30^Si and Mg/Si ratio across diverse meteorite types has been explained by equilibrium isotope fractionation between gaseous SiO and high temperature RLE rich condensate (forsterite, Mg_2_SiO_4_) in the solar nebula^35^. Since condensation of forsterite consumes half of nebular Si whereas the remaining half stays back in gaseous phase, it is postulated that equilibrium isotope fractionation between solid and gas should enrich forsterite condensates in heavier Si isotopes and fractionate the nebular gas to isotopically lighter δ^30^Si values by −1.59‰^35^. The removal of heavier forsterite and subsequent formation of enstatite grains from such gaseous reservoir is expected to enrich all enstatite silicates in significantly light Si isotopes relative to forsterite, which is not observed. Moreover, such a model invoking Si isotope fractionation associated with forsterite condensation predicts that enstatite chondrites with low Mg/Si ratios should have the lowest ^26^Mg/^24^Mg among all primitive meteorites. However, the recently published high precision Mg isotope study has reported that EC have the heaviest Mg isotope composition compared to any other anhydrous chondrites^22^, which is consistent with our Mg isotopes results (Table 2). Also, δ^30^Si does not appear to be strongly correlated with Mg/Si ratio across all planetary materials as Mg/Si of CC and OC are almost constant and the ratio decreases sharply with EL and EH chondrites^26^. Thus, Si and Mg isotope data of bulk enstatite chondrites and its phase separates indicate the dominance of other nebular or planetary scale processes in creating the observed δ^30^Si variations across solar system materials. In the following paragraphs, we use Si isotope data from a broad spectrum of meteorite types and micro-phase separates of EH3 chondrites along with thermodynamic constraints on condensation sequence of solids in oxygen-enriched and deficient nebular regions, to reconstruct the accretionary sequence of planetary bodies that originated in oxidized *versus* reduced conditions.

**Table 2:** Mg isotope composition of silicate and matrix fractions of EH3 chondrites analyzed in the same aliquot of EC-micro-phase separates where Si isotope analyses were performed.

Sample ID	Av. δ^26^Mg (‰)	2 SD	N
**Mg isotope standards**			
SRM-980 (Mg isotope standard)	−3.98	0.07	6*
BHVO-2	−0.23	0.06	7*
**Silicate grains**			
PCA 91461	−0.30	0.08	3
LAR 06252	−0.26	0.02	2
MIL 07028	−0.28	0.05	2
**Matrix grains**			
PCA 91461	−0.31	0.07	10
LAR 06252	−0.28	0.01	2
MIL 07028	−0.30	0.03	2
**Average δ**^**26**^**Mg EC (Silicate** + **Matrix)**	**−0.29**	**0.04**	21
**Literature data**			
BHVO-2 (Sikdar and Rai, 2017)	−0.26	0.08	44*
Bulk EC (Teng *et al*., 2010)	−0.29	0.06	17*
BSE (Teng *et al*., 2010)	−0.25	0.07	139*

The Mg isotope composition of external and terrestrial rock standards from this study and published data are provided for comparison. ‘N’ refers to the number of individual micro-milled phases where isotope analyses were performed. For standards and literature data, N* refers to the number of replicates.

### Si isotopes in unfractionated nebular reservoir

It is believed that EC like reduced planetary bodies were accreted in the inner regions of solar system with higher C/O ratio (~0.83) compared to solar value (~0.5)^43,44^. On the other hand, planetary bodies like Mars, Vesta, and OC-CC parent bodies originated at larger heliocentric distances in a more oxidizing environment^45^. In the oxygen rich conditions of solar nebula, Si always bonds with oxygen as silicate ion based on basic chemical unit SiO_4_^4−^. Due to the strict lithophile behavior of Si, nebular Si is expected to quantitatively condense into silicate phases of planetesimals originating at higher oxidation states. Hence, planetary bodies accreting in larger heliocentric distances are likely to avoid any major Si isotope fractionation associated with partitioning of Si between metal and silicate phases. The indistinguishable Si isotope composition between carbonaceous (Av. δ^30^Si_CC_ = −0.44 ± 0.07‰) and ordinary chondrites (Av. δ^30^Si_OC_ = −0.42 ± 0.05‰, Table S1) indicates that CC and OC (Av. δ^30^Si_CC-OC_ = −0.43 ± 0.06‰, This study + Literature data) have originated from an unfractionated Si isotope nebular reservoir. This is further supported from the absence of any systematic δ^30^Si differences between CV (−0.43 ± 0.06‰), CM (−0.47 ± 0.03‰) and ordinary chondrites (H (−0.43 ± 0.04‰), L (−0.42 ± 0.06‰), LL (−0.42 ± 0.07‰)). The Si isotope distribution among different types of CC and OC implies that the δ^30^Si of oxidized planetary materials is not largely dependent on the volume of chondrules or metal content^28,30^, except for certain degrees of alteration due to clay formation or aqueous activities.

The prevalence of identical silicon isotope reservoir at larger heliocentric distances is further evinced from the existence of Mars (δ^30^Si_SNC_ = −0.48 ± 0.13‰^30^) at 1.5 AU with approximately similar δ^30^Si as OC and CC (Av. δ^30^Si_CC-OC_ = −0.43 ± 0.06‰). Also, no statistically resolvable δ^30^Si differences exist between Howardites (−0.42 ± 0.02‰), Eucrites (−0.41 ± 0.04‰), and Diogenites (−0.42 ± 0.05‰); which are known to originate at varying depths of differentiated asteroid 4-Vesta^46^. Although we have not detected any Si isotope differences between mean HED and CC at the level of our analytical uncertainties, a former study has reported that silicate portion of HED parent body (represented by Eucrite and Diogenite) have slightly heavier δ^30^Si when bracketed relative to carbonaceous chondrites^32^. Despite presence of a well-defined metallic core, the lack of Si isotopic fractionation among different HED meteorites and possibly small δ^30^Si offset between HED and CC can be explained by formation of the Vestan core at relatively higher *f*O_2_ (ΔIW~−2 to ΔIW~−4)^32,47^ compared to very low oxygen fugacities of EC (IW−6 to IW−8)^4^. Since sequestration of a substantial amount of Si into metals is not possible in oxygen fugacities prevailing at HED accreting region, these planetary bodies likely have escaped any large-scale Si isotope fractionation associated with metal-silicate partitioning. Thus, HED clan of meteorites (Av. δ^30^Si_HED_ = −0.42 ± 0.04‰, This study) roughly preserves the unfractionated Si isotope composition of nebula, similar to carbonaceous and ordinary chondrites. A characteristic feature of condensation sequence of solids at solar C/O condition is the nearly identical condensation temperature of Fe-alloy and forsterite (major silicate of solar system) at 1357 K and 1354 K respectively^48^. Therefore, it is expected that metals and silicates of relatively oxidized nebular region were condensed and accreted simultaneously to form a homogeneous planetary body, which upon partial melting underwent internal differentiation to form the core with Si isotopes remaining least affected by metal-silicate partitioning.

### Si isotopes and the evolution of enstatite chondrites

Enstatite chondrites are nebular condensates that were formed under highly reducing and/or sulfurizing conditions^49^. The formation mechanism of reduced mineral assemblages in EC has been attributed to several processes such as multi-step fractionation involving the removal of refractory component, depletion of water in the solar nebula, equilibration with nebular gas at low- to intermediate temperatures of ~700–950 K^50^ and isolation of solids from solar gas^51^. It is largely believed that the unique mineralogy of EC is a result of condensation in a source region with low H_2_O/H_2_^25^ or high C/O ratio (0.83)^43,44,52^. However, trace element analyses and REE patterns in EH3 enstatite chondrites have been used to argue that the peculiarities of enstatite chondrites may not require a condensation sequence at high C/O ratio^53^ and could be rather described using sulfidation of ferromagnesian silicates or impact melting^54,55^. On the other hand, a subsequent study dealing with mineralogical, textural and nano-scale isotope studies has shown that EC have witnessed variable C/O ratio during the course of their evolution^56^. Therefore, the origin and evolution of these reduced meteorites has remained a subject of debate till date.

The most abundant phases of EC, i.e., enstatite silicates and Fe-Ni metal (representing ~50 wt% and 25 wt% of total sample^9^) were probably formed through condensation from a cooling nebular reservoir. Since vapor fractionation typically produces larger isotopic differences than any magmatic processes^22^, the wide range of Si isotope values preserved among distinct metal and silicate grains of EH3 chondrites at millimeter scale indicate that the isotope variations have perhaps survived from the earliest stages of nebular condensation established through gas-solid interaction processes. One of the major differences in the condensation sequence of solids at elevated C/O ratio against solar C/O lies on the appearance of silicates relative to metals. At C/O = ~0.83, the 50% condensation temperature of silicates and oxides get markedly depressed by ~200 K with the exception of metals such as Fe, whose condensation temperature is independent of the availability of free oxygen in the nebula^43^. Therefore, metals become relatively more refractory in reducing conditions leading to their earlier condensation compared to silicates. Being formed under highly reducing conditions, enstatite chondrites are known to incorporate 2–6% Si in solid solution with its metallic phases^40^. Formation of Fe-Si alloy generally takes place through substitution of Fe atoms by Si in the alloy structure and not just by mere partitioning into interstitial sites. Substitution of light elements such as Si or S for Fe is known to have a greater effect on the average bonding environment of the alloy^57^. Therefore, it is possible that lowering of Fe-Si bond strength associated with increasing Si content in Fe(Ni)-Si alloy structure could have led the light Si isotopes (^28^Si) to impinge the early-condensed reduced metals more rapidly relative to the heavier isotopes. This is consistent with ab-initio calculations, which suggest that equilibrium fractionation between Si in Fe-metal and SiO/ SiS gaseous reservoir is expected to enrich EC-metals in lighter Si isotopes^42^.

Since the stability field of liquids condensing from nebular gas lies in a restricted temperature-pressure range, Fe-Ni metals were most likely condensed directly as solids, thereby hindering the re-equilibration of metals with any subsequently formed solids or co-existing gases^26,58^. If true, an inevitable consequence of the incorporation of significantly light Si isotopes into metal condensates is alteration of the immediate surrounding silicate-dominating gaseous reservoir to progressively heavier Si isotopes. Such kind of isotope fractionation would imply that the solid (metal) condensates and silicates were condensed from a rather narrow region of reduced nebular reservoir and different phases of EC were formed in multiple steps from a nebular reservoir with evolving C/O ratios. With subsequent drop in nebular temperature, a new generation of solids composed mostly of silicates is expected to condense with fractionated Si isotope composition relative to CC-OC. This is further demonstrated by the following mass-balance calculations.

We assume that Av. δ^30^Si_CC-OC_ = −0.43 ± 0.06‰ (This study+Literature) represents the unfractionated Si isotope composition of solar nebula where Si was quantitatively condensed into silicate phases. Let ‘*f’* represent the fraction of total nebular silicon in the inner reduced region that was incorporated into metals of EC like planetary bodies. If δ^30^Si_metal_ = −6.94 ± 0.09‰ (the lightest δ^30^Si measured in EC-metal) and δ^30^Si_silicates_ = −0.27 ± 0.06‰ (the heaviest Si measured in silicate grain of the same meteorite) represent two end member composition of a continuum metal-silicate Si isotope fractionation process in inner solar nebula, the value of *‘f’* comes out to be 2.4 ± 0.05% from Eq. (1).

1 $$f\,\ast \,{\delta }^{30}S{i}_{{\rm{E}}{\rm{C}}{\textstyle \text{-}}{\rm{M}}{\rm{e}}{\rm{t}}{\rm{a}}{\rm{l}}}+(100-f)\,\ast \,{\delta }^{30}S{i}_{{\rm{E}}{\rm{C}}{\textstyle \text{-}}{\rm{S}}{\rm{i}}{\rm{l}}{\rm{i}}{\rm{c}}{\rm{a}}{\rm{t}}{\rm{e}}{\rm{s}}}=100\,\ast \,{\delta }^{30}S{i}_{{\rm{C}}{\rm{C}}{\textstyle \text{-}}{\rm{O}}{\rm{C}}}$$

Since the calculated value of *‘f’* lies well within the range of Si concentrations reported from EC metals (1.8–6%)^40,49^, it can be suggested that partitioning of light Si isotopes into metals containing at least 1.8% of total nebular Si can generate considerable Si isotope fractionation in reduced planetesimals.

However, even if we consider that significantly light Si isotopes were incorporated by 6% of total nebular Si, the average δ^30^Si of bulk EH3 chondrites still falls below δ^30^Si_CC-OC_, implying certain additional processes might have also fractionated Si isotopes in reduced nebular region apart from metal-silicate partitioning. It is emphasized that we have only measured the Si isotope composition of kamacite metals and enstatite silicates as their larger dimensions allowed us to micromill them more effectively compared to other phases (Perryite, Sinoite etc). Therefore, there remains a possibility that some other lighter/heavier Si isotope enriched components could have remained ‘unaccounted’ within EC. The representatives of EC-silicates were micro-milled through point scan mode of drilling from the interior most central part of large silicate grains to avoid sampling of metals or sulfides from surrounding rims. The metals, on the other hand, were too small in size (<100 µm) to micro-mill without completely avoiding dilution by heavier silicates. Hence, a larger Δ^30^Si_metal-silicate_ value in EC is expected, which might have an affect on the total mass balance as given by Eq. 1.

### Origin of Earth’s Si isotope composition

The remarkable similarity between EC and BSE for several isotope systematics has been used to suggest that Earth accreting materials were originated in a narrow region of inner proto-planetary disk with approximately uniform isotopic composition as enstatite chondrites^5,59^. However, the chemical composition of EC is not compatible with terrestrial mantle and hence it is increasingly difficult to form the Earth with EC building blocks. To test the involvement of EC like planetary bodies in Earth’s accretion from Si isotope perspective, here we discuss the case of metal-silicate isotope equilibration at conditions relevant to the base of magma-ocean (i.e., 3000 K and 40 GPa).

First we assume that bulk Earth has enstatite chondritic composition (δ^30^Si_EC_ = −0.61 ± 0.11‰) and core formation of the Earth occurred with full metal-silicate equilibration during magma ocean stage. Upon considering the experimentally derived temperature-dependent metal-silicate silicon isotope fractionation factor (i.e., Δ^30^Si_silicate-metal_) of ((7.45 ± 0.41‰) × 10^6^/T^2^)^60^, δ^30^Si_BSE_ = −0.29 ± 0.08‰^33^, Si fraction of BSE = 0.212, and mass fraction of core = 0.32^28,30,31^, the observed Si isotope offset between bulk EC and BSE requires the presence of 27.3 ± 0.22% Si in Earth’s core for EC-Earth model, which is an unrealistic scenario. Therefore, either the assumption that heavier Si isotope composition of BSE is a result of complete equilibration between metallic core of larger impactors and the mantle may not be valid or bulk enstatite chondrites cannot form a significant component of Earth’s precursor material. The case of no large-scale ‘isotope’ re-equilibration between Si-rich metal and silicate mantle would require that Earth has preserved Si isotope heterogeneity established through metal-silicate Si isotope fractionation at nebular settings. However, more than 36% of equilibration is necessary to account for siderophile element abundances and Hf-W composition of Earth’s mantle^61,62^. Therefore, merging of metals without any equilibration with the surrounding silicates is an unlikely situation.

**Figure 3:**
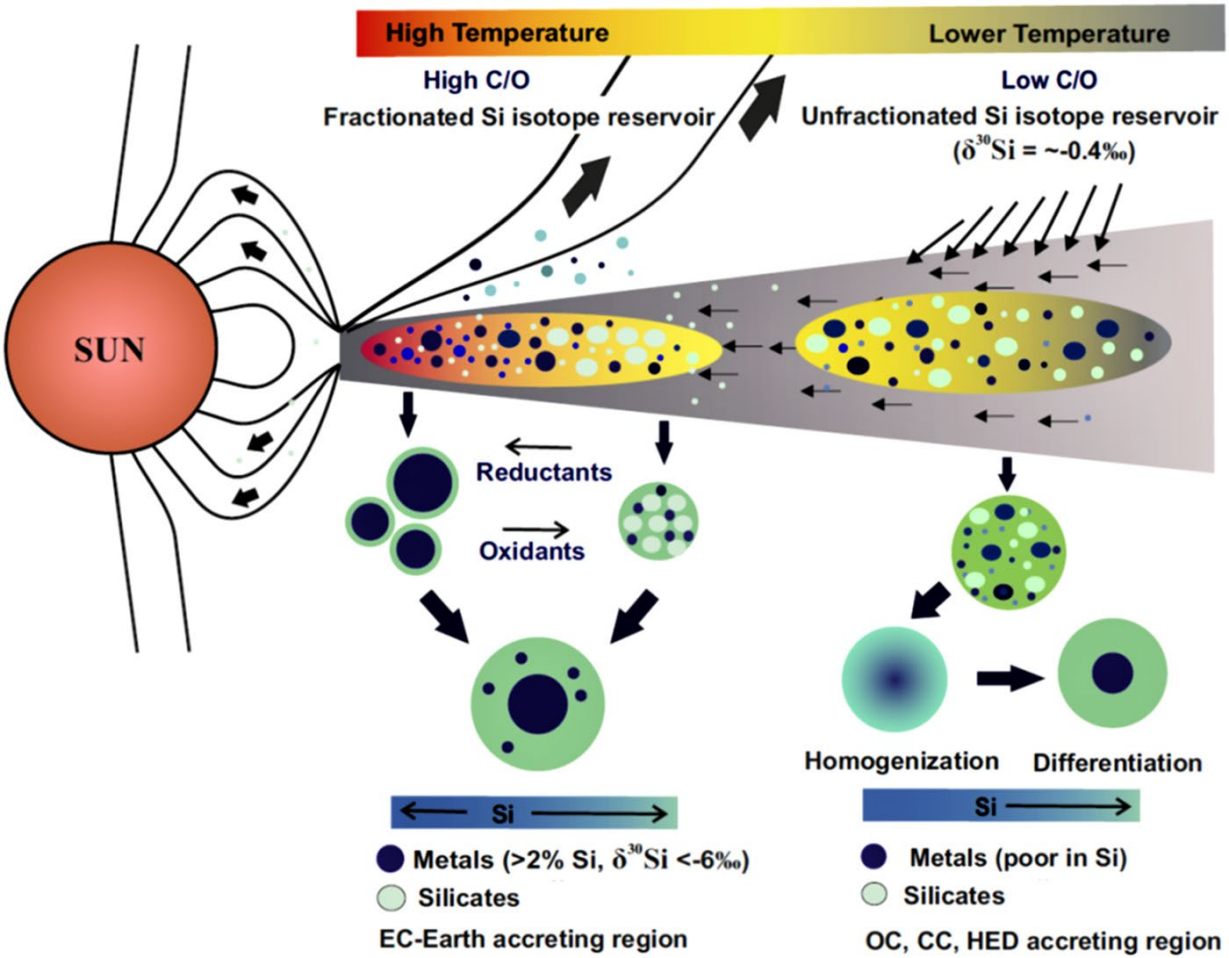
Schematic diagram showing the accretion and core-forming scenario of different meteorite parent bodies and the Earth. OC, CC, SNCs and HEDs were formed in oxidized conditions, farther away from Sun where Si was quantitatively condensed into silicate phases. Under oxygenated conditions, metals and silicates condensed simultaneously and accreted together to form a homogeneous planetary body, which later underwent density segregation to form the core with negligible Si isotope fractionation. Contrary to this, EC parent bodies were formed closer to the Sun in reduced nebular environment where metals were condensed earlier than silicates and Si isotopes underwent metal-silicate fractionation as a result of gas-solid interaction processes. BSE might have partly inherited its Si isotope composition from Earth forming raw materials that followed a similar sequence of Si isotope evolution as enstatite chondrite (EH3) parent bodies.

The magnitude of Si isotope composition determined within micro-phase separates of EH3 chondrites indicates that extensive Si isotope fractionation has occurred in reduced planetary bodies irrespective of the size of their parent planet and high temperature-pressure conditions attainable during core formation. This would imply that the fractionated Si isotope signature of BSE is not a unique consequence of high temperature-pressure conditions attainable during core-mantle equilibration of a fully-grown planet such as the Earth. Although enstatite chondrites might not have been directly involved in Earth’s accretion, the close δ^30^Si between EH3-silicates and BSE suggests that parent bodies of EH3 chondrites and Earth forming embryos had undergone more or less similar sequence of Si isotope evolution at least during initial stages of planet formation. As such, terrestrial mantle might have partly inherited its isotope signature from Earth-forming raw materials that had already witnessed metal-silicate Si isotope fractionation at reduced nebular environment prior to accretion onto the Earth. Given this, we speculate that enstatite chondrites are a potential analogue planetary material to understand early stages of Earth’s accretion, when prevailing reduced conditions allowed substantial incorporation of Si (or S) into the planet’s metallic core although they cannot represent the sole building blocks of the Earth. The corollary of the above statement is that the rather narrow Earth accreting region must have been gradually oxidized as the planet continued to grow or Earth might have accreted raw materials from oxidized nebular reservoir at later stages of its accretion, which subsequently added FeO to terrestrial mantle during multiple episodes of impacts between impactors and the embryonic Earth. A schematic diagram depicting the accretion and core-formation sequence of various meteorite parent bodies based on Si isotopic constrains is illustrated in Fig. 3.

### Mg isotopes and vapor loss in Earth forming planetesimals

Being a moderately refractory element, the isotopes of Si can potentially fractionate as a result of vapor loss of lighter isotopes during planetary collisions. Hence the non-chondritic Si isotope composition of BSE has been also attributed to impact-induced volatilization from growing planetesimals^22,34^. Since differences in volatility of species was one of the major criteria that governed isotope fractionation in the early solar nebula, an ideal system to look for the effect of vaporization in generating any Si isotope fractionation on planetary surfaces would be Mg isotopes as Si shares comparable 50% condensation temperature with Mg in both solar and elevated C/O environments (T_c_ of Si and Mg are 1310 and 1336 K respectively^48^). The effect of partial loss of lighter isotopes during evaporation is best documented in calcium aluminium-rich refractory inclusions (CAIs), which are natural evaporative residues with heavy isotope enrichment of both Si and Mg by several per mill relative to terrestrial composition^63^.

To understand the influence of impact induced vaporization in generating any Si isotope fractionation of BSE, we have carried out Mg isotopic analyses notably in the ‘same aliquot’ of silicate and matrix fractions of EH3 chondrites where Si isotope analyses were performed following a newly developed chromatographic protocol^64^. Despite the presence of significant Si isotope heterogeneity, resolvable Mg isotope differences are not observed among different micro-phases of EC. Moreover, the mean Mg isotope composition of EC-silicate and matrices (Av. δ^26^Mg_silicate+matrices_ = −0.29 ± 0.04‰) overlaps with the reported Mg isotope composition of bulk EC (δ^26^Mg_EC_ = −0.29 ± 0.06‰^65^).

**Figure 4:**
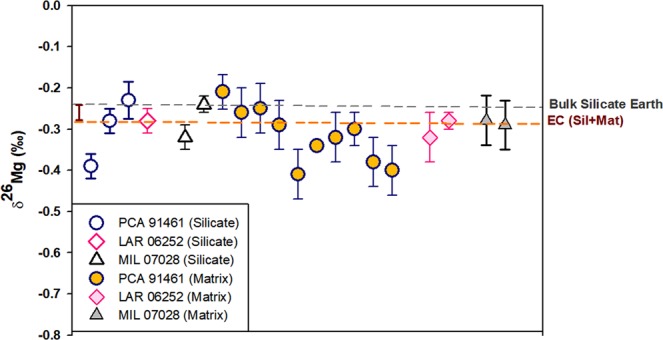
Mg isotope data from silicate and matrix fractions of EH3 chondrites obtained from the same aliquot of samples used for Si isotope analyses. The two horizontal dashed lines represent the established δ^26^Mg of Bulk Silicate Earth^65^ and the average δ^26^Mg of micro-phase separates of enstatite chondrites determined in this study. Z-test suggests statistically significant Mg isotope differences exist between δ^26^Mg of BSE and Enstatite chondrites.

High precision Mg isotope analyses of bulk meteorites have shown that the mean δ^25^Mg of enstatite chondrites are around 0.013‰ lower than that of differentiated bodies such as the Earth whereas OC and CC display larger Mg isotope offset relative to BSE^22^. In agreement with the former study, we have also observed that the majority of Mg isotope data on silicate and matrix fractions of EH3 chondrites rather lies on isotopically lighter side of BSE, (δ^26^Mg_BSE_  = −0.25 ± 0.07‰^65^), Fig. 4. Z-test was used to determine if any statistically significant Mg isotope differences exist between δ^26^Mg of BSE and Enstatite chondrites (data from Teng *et al*., 2010 and this study). It was found that the calculated z value (3.579) exceeds the tabulated z-critical two-tail value (1.959), which suggests that the means of δ^26^Mg_BSE_ and δ^26^Mg_EC(Sil+Mat)_ are significantly different at 95% confidence level. Considering that EC like planetary bodies and the Earth were originated from a narrow isotope reservoir, the observed Mg isotope difference between EC and BSE requires about 15% vapor loss of light Mg isotopes from terrestrial magma ocean. Since the extent of vaporization of Mg is generally lesser compared to Si^22^, planetary volatilization should produce lower Mg isotope fractionation compared to Si isotopes, not more. Therefore, based on our Mg isotope data, it can be suggested that preferential loss of light Si isotopes from growing planetesimals during impact-induced vaporization could have further configured the Si isotope signature of BSE. Thus, we conclude that the fractionated δ^30^Si of BSE relative to ordinary/carbonaceous chondrites likely reflects the combined consequences of processes such as planetary/nebular metal-silicate Si isotope partitioning under reduced conditions and vapor loss of lighter Si isotopes from the Earth accreting region.

## Methodology

### Electron Probe Micro Analyzer

Polished mounted sections of enstatite chondrites were analyzed using a CAMECA SX100 EPMA to determine major-element composition and mineralogical characterization of various chondritic phases. Back Scattered Electron images of different areas of the sample were captured and stitched to form a collage of the whole meteorite surface. Emphasis was given to search for least altered and impurity free phases. Quantitative chemical analyses of target phases were determined using wavelength dispersive spectrometers (WDS) operated at 15 kV accelerating voltage and 15 nA beam current. Calibration of spectrometers for measuring counts of Si, Mg and Fe was done using diopside (CaMgSi_2_O_6_), olivine (Mg_2_SiO_4_) and troilite (FeS_2_) respectively. The composition of enstatite (MgSiO_3_) in EC was found to be, more or less, uniform among silicates, chondrules and matrix with ~60% SiO_2_ and ~40% MgO respectively. Kamacite and perryite are the two metallic phases with high abundance of silicon, containing 2–3 and 11–15wt% Si respectively.

### New Wave Micromill

Prior to micro-milling, carbon coating on the polished samples was removed. The locations of pre-characterized metallic and silicate phases of chondrites were first identified under optical microscope attached to New-Wave Micro-mill machine. Computer assisted micro-milling of target phases were carried out under microscope using Tungsten drill bit having a cross section of 100 µm. 20–50 µl of ultrapure water (MQ) was added on the target location to avoid scattering of the powdered mineral dusts while drilling. The wet sample slurry was collected with a micro-pipette into a pre-weighed Savillex^TM^ Teflon vial, where it was eventually digested. A microbalance was used to weigh the samples after careful evaporation of water. To avoid cross contamination among different chondritic phases, the mounted sample and drill bits were cleaned with ethanol and wiping with MQ after each micromilling session. Every possible measure was taken not to sample sulfides while specifically drilling the representative samples of metals and silicates (*not matrices*), because presence of sulfur has been reported to induce artificial Si isotope fractionation^66^. Based on size of the target phases, point or line scan mode of drilling was applied whereas raster scanning was used to drill the representative samples of matrices and bulk meteorites.

### Sample digestion and column chromatography

To avoid cross-contamination, sample loss and blank contribution while processing the small volume of micro-milled EC phase separates, the samples were digested in the same Teflon vial where it was stored after micromilling using a modified alkali digestion technique^64^. Approximately 0.05−0.5 mg of chondritic samples were mixed with ~30–50x mg sodium hydroxide monohydrate (NaOH) flakes (Suprapure from Merck), where ‘x’ represents weight of the sample. The sealed vial was heated at ~250 °C on a hot plate for ~36 hours. After substantial cooling, the resultant mixture was dissolved using HCl and ultra-pure water. The pH of solution prior to loading onto columns was monitored. Digested rock solutions were ultrasonicated and left undisturbed to equilibrate for at least 18 hours. During the entire digestion procedure, extreme care was taken to inhibit the formation of any crystals since adsorption of Si on Fe hydroxides can induce a shift in silicon isotope fractionation.

Chromatographic purification of Si was carried out using 2 ml BioRad cation exchange resin AG 50W-X8 (200–400 mesh) in H^+^ form. Briefly, samples containing ~3–8 µg of Si were loaded on the resin and Si was eluted using 10 ml Milli-Q. For metal enriched phases, column chromatography was repeated at least 4–5 times until the the Fe-Ni contents of purified samples measured after column chemistry reached below instrumental blank. A larger volume of MQ water was usually required to recover Si quantitatively from metal enriched phases. Recovery of Si from both fusion and ion chromatography stage was >98 ± 5%. Simultaneous purification of Mg from the same aliquot of meteorite phases (matrices and silicates) used for Si purification was carried out following a newly established chromatographic protocol^64^. The total procedural blank was <10 ng for both Si and Mg isotope measurements.

### Thermo Neptune MC-ICPMS

High precision Si and Mg isotope analyses were carried out using Thermo Scientific Neptune Plus MC-ICPMS at PRL, Ahmedabad (India). Samples were introduced via APEX-Q attached with Nefion micro porous membrane desolvator. The semi-dry plasma condition achieved through the above mentioned setup reduces the isobaric effects of interfering molecular species by minimizing introduction of H_2_O, CO_2_, O_2_ and N_2_ into the plasma. To resolve polyatomic interferences, MC-ICPMS was operated in high-resolution mode with mass resolving power of >8000 and isotope measurements were made on the low mass side of the peak plateau. Instrument mass bias was corrected via standard sample bracketing technique whereby NBS-28 and DSM-3 were used as bracketing standard for Si and Mg isotope analyses respectively. The isotope measurements of one block of Si and Mg isotope analysis consisted of 50 cycles of data acquisition with an integration time of 8.389 seconds per cycle. The details of instrumental set up used for Si and Mg isotope analyses can be found elsewhere^64^.

## Supplementary information

Si-Mg isotopes in enstatite chondrites and accretion of reduced planetary bodies.

## Data Availability

Most of the data generated during this study are included in this published article and its Supplementary Information file.
