# Supplementary material for: Si-Mg isotopes in enstatite chondrites and accretion of reduced planetary bodies
Source: Sci Rep. 2020 Jan 27;10:1273. doi: 10.1038/s41598-020-57635-1 (PMC6985146; doi:10.1038/s41598-020-57635-1)
Supplement: Supplementary file 1 — Si-Mg isotopes in enstatite chondrites and accretion of reduced planetary bodies. [file 41598_2020_57635_MOESM1_ESM.pdf]

Si-Mg isotopes in enstatite chondrites and accretion of reduced planetary bodies

Jinia Sikdar[a]* and Vinai K. Rai[a,b]

*Corresponding author: jinia@prl.res.in

[a]Physical Research Laboratory, Ahmedabad 380009, India

[b]School of Earth and Space Exploration, Arizona State University, Tempe, AZ 85281, USA

Supplementary Table S1: Si isotope data of secondary rock standards and bulk meteorite samples measured by sample-standard bracketing method against NBS-28. The sampling procedure used for obtaining bulk rock representative of individual meteorite types is mentioned adjacent to the sample ID. 'N' denotes the number of repeat measurements of individual samples. For averages, the total number of meteorites analyzed in each group is represented by 'N*'. Literature values are provided at the end to compare and establish the robustness of our analytical and isotope measurement technique.

Meteorite Name/ Sample ID	Meteorite type	Sampling procedure	δ^{30}Si (‰)	2 SD	N
Diatomite			1.22	0.09	30
Diatomite (Armytage et al., 2011)			1.23	0.15	400
BHVO-2			−0.27	0.08	24
BCR-2			−0.26	0.09	7
**Carbonaceous Chondrite**					
Y-86751	CV3	Rock powder	−0.39	0.08	2
Y-74662	CM2	Rock powder	−0.46	0.05	3
EET 96029	CM2	Raster Micro-mill	−0.48	0.05	3
GRA 06101	CV3	Raster Micro-mill	−0.45	0.09	2
ALH 84028	CV3	Raster Micro-mill	−0.44	0.06	3
Carbonaceous chondrite average	-	-	**−0.44**	**0.07**	**5***

**Ordinary Chondrite**					
WSG 95300	H 3.3	Raster Micro-mill	−0.45	0.06	3
ALHA 77299	H 3.7	Raster Micro-mill	−0.41	0.06	2
KASAULI	H4	Rock powder	−0.44	0.05	3
Y-980448	L3	Rock powder	−0.40	0.07	3
Y-981274	L3	Rock powder	−0.38	0.07	1
QUE 97008	L3.0	Raster Micro-mill	−0.44	0.08	2
ALHA 77216	L 3.7-3.9	Raster Micro-mill	−0.41	0.10	3
JODIYA	L5	Rock powder	−0.41	0.02	2
ALHA 83007	LL3.2/3.5	Raster Micro-mill	−0.42	0.07	1
ITAWA BHOPJI	L 3-5	Rock powder	−0.47	0.05	2
Ordinary chondrite average	-	-	**−0.42**	**0.05**	**10***
**Enstatite meteorites**					
PCA 91461	EH3	Raster Micro-mill	−0.64	0.12	8
LAR 06252	EH3	Raster Micro-mill	−0.62	0.03	3
MIL 07028	EH3	Raster Micro-mill	−0.65	0.06	2
Y-691	EH3	Rock powder	−0.66	0.09	4
ALHA 81021	EL6	Raster Micro-mill	−0.55	0.04	2
LAR 04316	Aubrite	Raster Micro-mill	−0.53	0.04	2
Enstatite chondrite-Aubrite average	-	-	**−0.61**	**0.11**	**6***
**HEDs**					
LOHAWAT	Howardite	Rock powder	−0.41	0.05	2
QUE 97001	Howardite	Rock powder	−0.43	0.07	2
EET 87503	Howardite	Rock powder	−0.42	0.03	2
EET 87542	Eucrite (Brecciated)	Rock powder	−0.39	0.02	3
ALHA 76005	Eucrite (Polymict)	Rock powder	−0.42	0.05	2
SHALKA	Diogenite	Rock powder	−0.44	0.03	2
LAP 03569	Diogenite	Rock powder	−0.43	0.04	2
MIL 07001	Diogenite (Olivine)	Rock powder	−0.39	0.07	3
HED meteorite clan average	-	-	**−0.42**	**0.04**	**8***
**LITERATURE DATA**					
BSE (Savage et al., 2010)	Bulk Silicate Earth	-	−0.29	0.08	35*
BSM (Armytage et al., 2012)	Bulk Silicate Moon	-	−0.29	0.08	24*
Ang. (Dauphas et al., 2015)	Angrite	-	−0.22	0.07	4*
CC (Armytage et al., 2011)	CC	-	−0.48	0.10	8*
OC (Armytage et al., 2011)	OC	-	−0.46	0.06	8*
OC-CC (Zambardi et al., 2013)	Av. OC-CC	-	−0.46	0.07	9*
**Average CC-OC (This study+Lit)**	**Av. OC-CC**	-	**−0.43**	**0.06**	**40***
Ureilite (Armytage et al., 2011)	Ureilite	-	−0.47	0.12	4*
HED (Pringle et al., 2013)	Vestan meteorites	-	−0.43	0.11	14*

SNC (Armytage *et al.,* 2011)	Martian meteorites	-	−0.48	0.13	5*
EC (Armytage *et al.,* 2011)	EC	-	−0.63	0.07	3*
(Savage and Moynier, 2013)	EH	-	−0.77	0.08	6*
(Savage and Moynier, 2013)	EL	-	−0.59	0.09	7*
Aubrite (Savage and Moynier, 2013)	Aubrite		−0.60	0.11	5*

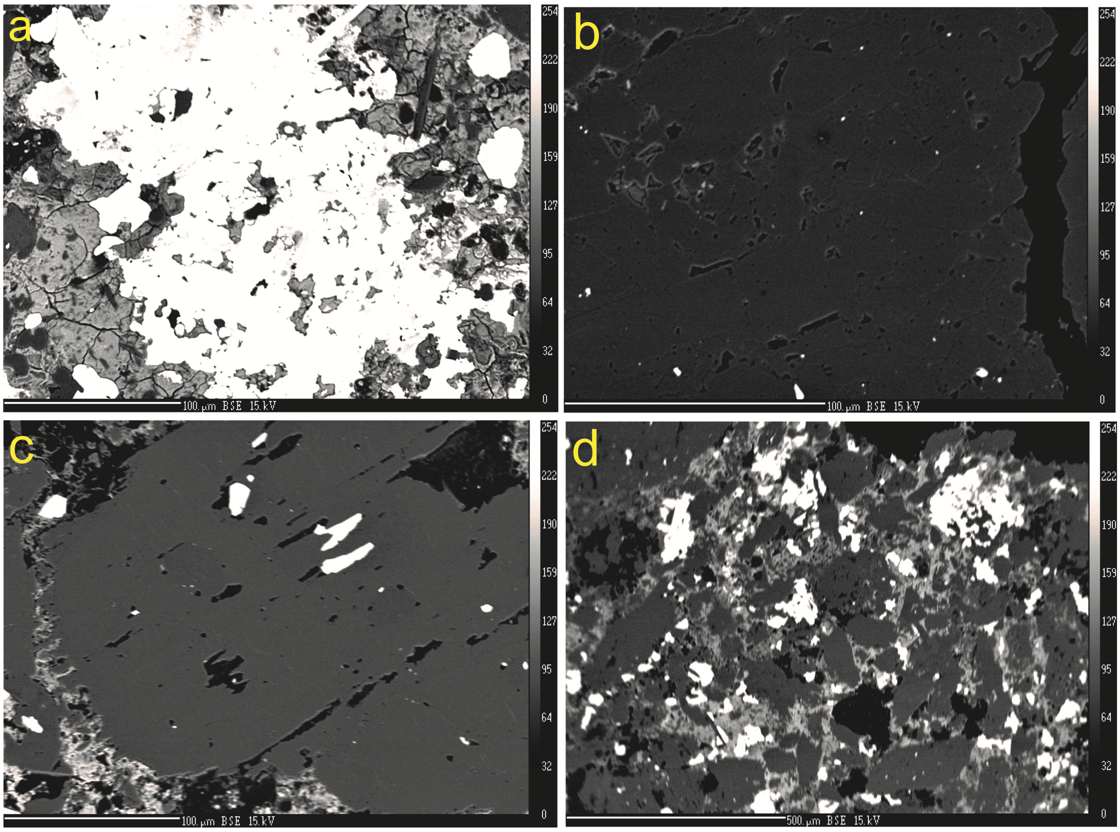

Supplementary Figure S1: Back-Scattered-Electron images of a typical a) metal, b-c) silicate, and d) matrix fractions of EH3 chondrites considered for Si and Mg isotope analyses in the present study. The boundaries of different phases are sharp, which indicates the unequilibrated nature of the studied meteorites. Presence of disseminated metals within silicates and vice-versa is evident in a micron scale.

REFERENCES

1. Armytage et al. Si isotopes in meteorites and planetary core formation. *Geochim Cosmochim Acta* **75**, 3662–3676 (2011)
2. Sikdar J. & Rai, V. K. Simultaneous chromatographic purification of Si and Mg for isotopic analyses using MC-ICPMS. *J. Anal. At. Spectrom.* **32**, 822-833 (2017)
3. Savage et al. Silicon isotope homogeneity in the mantle. *Earth and Planetary Science Letters*, **295**, 139–146 (2010)
4. Armytage et al. Silicon isotopes in lunar rocks: implications for the Moon's formation and the early history of the Earth. *Geochimica et Cosmochimica Acta*, **77**, 504–514 (2012)
5. Dauphas et al. Planetary and meteoritic Mg/Si and δ30Si variations inherited from solar

nebula chemistry. *Earth and Planetary Science Letters* **427**, 236-248 (2015)
6. Zambardi et al. Silicon isotope variations in the inner solar system: Implications for planetary formation, differentiation and composition. *Geochim Cosmochim Acta* **121**, 67–83 (2013)
7. Pringle et al. Redox state during core formation on asteroid 4-Vesta. *Earth Planet Sci Lett* **373**, 75-82 (2013)
8. Savage, P. S. & Moynier, F. Silicon isotopic variation in enstatite meteorites: Clues to their origin and Earth forming material. *Earth Planet Sci Lett* **361**, 487–496 (2013)
